# Giant Nephrothoracic Abscess: A Misleading Disease, a Surgical Challenge, and an Unexpected Complication

**DOI:** 10.1155/2014/513579

**Published:** 2014-06-26

**Authors:** Peter Kronenberg, Bruno Graça, Manuel Ferreira Coelho

**Affiliations:** ^1^Hospital Prof. Doutor Fernando Fonseca, 2720-276 Amadora, Portugal; ^2^Hospital dos Lusíadas, 1500-458 Lisbon, Portugal

## Abstract

A rare case of perinephric abscess with unilateral secondary pulmonary involvement that was further complicated by spillover of purulent content into the contralateral lung is reported here. Its diagnosis, treatment, and evolution are described and discussed along with certain features of nephropulmonary fistulas. The diagnosis of these abscesses is difficult, largely because of the paucity of primary symptoms and the frequent presence of misleading secondary symptoms. Deceptive cases like this one highlight the importance of its contemplation in every physician's differential diagnosis.

## 1. Introduction

Perinephric and retroperitoneal abscesses are infrequent clinical entities. Concomitant thoracic involvement is even rarer: very few cases have been described worldwide [1–3]. Since their diagnosis is difficult, a rare case of a perinephric abscess that involved an even rarer complication is described here.

## 2. Case Presentation

**Figure 1:**
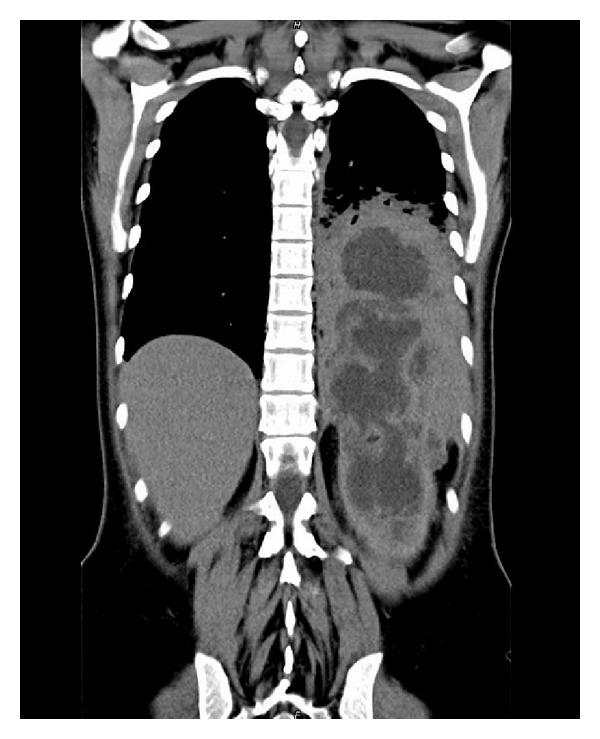
CT-scan revealing a large perinephric abscess with subphrenic extension and perforation of the diaphragm, causing a gigantic empyema which occupied the lower two thirds of the left hemithorax, totaling a 30 cm large abscess on its largest axis.

**Figure 2:**
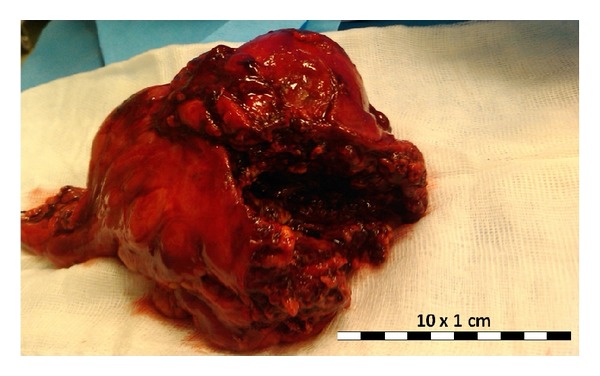
Total nephrectomy specimen revealing an altered renal anatomy due to parenchymatous degeneration.

**Figure 3:**
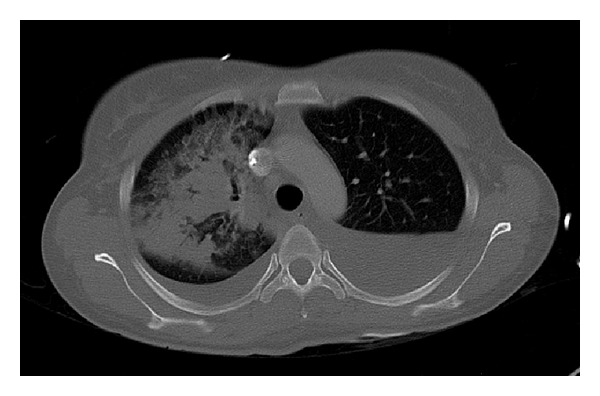
CT-scan showing* de novo* consolidation of the right lung, 24 hours after surgery.

**Figure 4:**
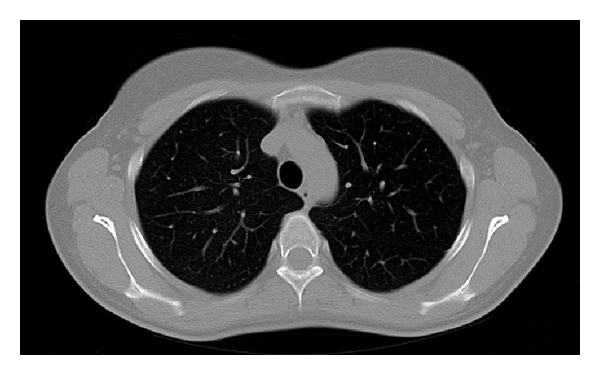
CT-scan of the same region depicted in Figure 3, three months later, showing complete remission of the right component of the bilateral pneumonia.

A 31-year-old female patient developed left lumbar pain, light fever, and intermittent hematuria. She had a previous history of ureteroscopic stone removal and recent childbirth. Several days after symptom onset, she consulted her family physician who suspected a urinary tract infection and prescribed oral antibiotics. After 5 days of symptomatic aggravation with persisting fever, back pain, and a recent productive cough with green-colored sputum, she came to our hospital. She presented with clinical and laboratory features of sepsis, with low hemoglobin (7.3 g/dL), leukocytosis (19800/*μ*L), high C-reactive protein (25.3 mg/dL), and leukocyturia. Simple computer tomography (CT) revealed a left pleural effusion. An enlarged left kidney was also observed but its significance was not appreciated at that time. Empirical antibiotic treatment (gentamicin) was initiated and two blood transfusions were given. Forty-eight hours later, the fever still persisted, the laboratory infection features remained high, and contrast-enhanced CT revealed a large perinephric abscess with a subphrenic extension perforating into the thoracic cavity, thus diagnosing a nephropleural fistula. This caused an empyema that occupied the lower two-thirds of the left hemithorax (Figure 1). A small calculus at the ureteropelvic junction was also present. A urological evaluation was requested and was swiftly followed by total nephrectomy (Figure 2) through the 10th intercostal space. A small diaphragm perforation was identified. Due to momentary unavailability of a chest surgeon the urologic surgeons proceeded with empyema drainage, digital decortication, and thorough cleansing. Since the diaphragm perforation was small and the surrounding tissue too friable, no attempt was made to repair the small perforation. Passive lumbar and active chest drainage tubes were placed. The antibiotic treatment was changed to a combination of piperacillin, tazobactam, and metronidazole. Twenty-four hours later, the patient presented with widespread bilateral lung infiltrates on X-ray and CT (Figure 3), especially on the right side, consequently admitting the likelihood of an undiagnosed nephrobronchial fistula component. She developed a bilateral pneumonia that required an 8-day stay in the intensive care unit and additional respiratory kinesiotherapy. However, mechanical ventilation was not needed. Two days after surgery, the chest surgeon who began following the patient postoperatively performed an additional CT-guided percutaneous drainage of the persistent residual empyema. Intrathoracic pus cultures revealed the presence of* Proteus mirabilis*. The patient made a full recovery with normalization of all laboratory values and improvement of the pulmonary radiological features. She was discharged at day 12. Three months later, residual pulmonary changes on follow-up imaging studies were not observed (Figure 4).

## 3. Discussion

Perinephric abscesses are rare and are difficult to diagnose unless the physician considers them during the differential diagnosis. However, their diagnosis may become easier if one understands the routes through which these abscesses develop (which include hematogenous dissemination, ascending urinary tract infection, or* via *contiguity), their risk factors, and their clinical features [2, 4, 5].

Recognized risk factors for perinephric abscesses are lithiasis, urological manipulation, and pregnancy, all of which our patient had in the past. Other risk factors are diabetes, immunosuppressive conditions, intravenous drug abuse, skin infections, any cause of urinary stasis, malignancy, and prolonged antibiotic use [2, 6, 7].

Due to the insidious nature of the disease and, sometimes, the paucity of symptoms, patients tend not to seek medical attention until later. Common presenting symptoms are fever (64–94% of patients) and pain (70–87%), predominantly in the flank or abdomen. Other frequent symptoms are nausea and vomiting (30–64%), weight loss (15-16%), and chills (9–40%); however, urinary tract symptoms, such as pollakiuria and dysuria, have only been reported in 6–12% of cases [2, 7]. When distant symptomatic complications arise, the often scarce renal symptoms may be overlooked or erroneously attributed to the secondary aggravation. As a result, most tests and clinical judgments focus on the secondary aggravation and the primary disease is not detected. This is problematic because this condition should be diagnosed as early as possible. This is underscored by the fact that patients with undiagnosed perinephric abscess who are admitted to medicine wards have 3-fold higher mortality rates than patients who are admitted to a surgical ward [8].

Patients with secondary pulmonary involvement (such as our patient) may present with a productive cough, pleurodynia, or dyspnea, and, in some cases, even a urine-like taste in the mouth due to a nephrobronchial fistula. Tachypnea, decreased breath sounds, and diminished resonance or dullness on thoracic percussion on the affected pulmonary side may also be present [1–3, 9].

Laboratory findings include leukocytosis (75–93%) and anemia (in 40% of patients, the hemoglobin levels are below 10 g/dL). Up to 66% of patients have leukocyturia, bacteriuria, and signs of hematuria. Thus, a normal urinalysis will not exclude a perinephric abscess [6–8].

In the past,* Staphylococcus aureus* was the most common causative organism (up to 80% of cases). However, more recent cases involve enteric gram-negative agents such as* Escherichia coli* (14–63%),* Klebsiella pneumoniae* (5–25%),* P. mirabilis* (5–21%), and* Pseudomonas aeruginosa* (5–11%) [6, 8]. In patients with nephropulmonary fistulas, the causative organisms are similar:* E. coli* and* P. mirabilis* again predominate [3, 9]. If suspicious bacterial species such as* Proteus* are found in pleural liquids or respiratory secretions, associated renal disease should be excluded [9].

Only contrast-enhanced CT allowed us to correctly identify the disease and link it to its associated complications. This was because contrast-enhanced CT allows evaluation of not only the urinary tract but also all other retroperitoneal and intra-abdominal organs and systems at the same time. This results in a well-established diagnostic accuracy of over 95% for many diseases [6, 7]. As a result, contrast-enhanced CT is the diagnostic imaging tool of choice. However, although they are not as accurate, ultrasonography or even simple X-rays can detect fluid collections, masses, or diaphragmatic anomalies, thus helping physicians to orient themselves [5, 6, 8].

Of the different treatment options that are available for perinephric abscesses, the open surgical approach bears the best results (98% versus 60% for percutaneous drainage), especially in multiloculated abscess cases [10]. Although nephron-sparing attitudes are more desirable, in case of widespread damage to the kidney parenchyma and severe septic conditions, nephrectomy is justified, such as in our case [2, 6, 7]. If the patient is too unstable for anesthesia, prompt percutaneous drainage should be attempted while postponing the open procedure for a later, more stable, setting [6]. Conservative treatment with antibiotics alone is very controversial: in some series, this approach is associated with a 100% mortality rate [5, 7].

Empirical broad-spectrum antibiotic coverage of gram-negative and -positive organisms is usually prescribed. Combinations of aminoglycosides (gentamicin or tobramycin) and beta-lactamic agents that also target* Staphylococci* (ampicillin, cefazolin, oxacillin, nafcillin, cephalothin, and cephapirin) are generally used. These treatments are generally adjusted according to positive cultures and given for 2-3 weeks after drainage [7].

Caution is advised if nephropulmonary fistulas are present or even suspected: what initially seemed to be a nephropleural fistula revealed itself as a nephrobronchial fistula with spillover of purulent material into the other lung in lateral decubitus during the surgical procedure. The positive pressure of the mechanical ventilation then forces the purulent material into the bronchioles and alveoli, thereby contaminating the contralateral lung and worsening an already serious pulmonary involvement. Moreover, the bronchial fistula component could only be identified postoperatively by its severe consequences. This condition is known as “lung down syndrome” [1]. The use of double lumen endobronchial tubes during the open procedure can prevent spillover from one lung to the other. In the rare event of thoracic involvement, it is essential that good surgical drainage and cleansing of the two anatomic regions be performed. If a thoracic surgeon is not available, the urologist must be sufficiently skilled to offer the best surgical management in such cases.
